# QTL Analysis and Fine Mapping of a Major QTL Conferring Kernel Size in Maize (*Zea mays*)

**DOI:** 10.3389/fgene.2020.603920

**Published:** 2020-11-27

**Authors:** Guiying Wang, Yanming Zhao, Wenbo Mao, Xiaojie Ma, Chengfu Su

**Affiliations:** ^1^College of Agronomy, Qingdao Agricultural University, Qingdao, China; ^2^Shandong Provincial Key Laboratory of Dryland Farming Technology, Qingdao Agricultural University, Qingdao, China

**Keywords:** candidate gene, maize, fine mapping, kernel size, NILs

## Abstract

Kernel size is an important agronomic trait for grain yield in maize. The purpose of this study is to map QTLs and predict candidate genes for kernel size in maize. A total of 199 F_2_ and its F_2__:__3_ lines from the cross between SG5/SG7 were developed. A composite interval mapping (CIM) method was used to detect QTLs in three environments of F_2_ and F_2__:__3_ populations. The result showed that a total of 10 QTLs for kernel size were detected, among which were five QTLs for kernel length (KL) and five QTLs for kernel width (KW). Two stable QTLs, *qKW-1*, and *qKL-2*, were mapped in all three environments. Three QTLs, *qKL-1, qKW-1*, and *qKW-2*, were overlapped with the QTLs identified from previous studies. In order to validate and fine map *qKL-2*, near-isogenic lines (NILs) were developed by continuous backcrossing between SG5 as the donor parent and SG7 as the recurrent parent. Marker-assisted selection was conducted from BC_2_F_1_ generation with molecular markers near *qKL-2*. A secondary linkage map with six markers around the *qKL-2* region was developed and used for fine mapping of *qKL-2.* Finally, *qKL-2* was confirmed in a 1.95 Mb physical interval with selected overlapping recombinant chromosomes on maize chromosome 9 by blasting with the Zea_Mays_B73 v4 genome. Transcriptome analysis showed that a total of 11 out of 40 protein-coding genes differently expressed between the two parents were detected in the identified *qKL-2* interval. GRMZM2G006080 encoding a receptor-like protein kinase FERONIA, was predicted as a candidate gene to control kernel size. The work will not only help to understand the genetic mechanisms of kernel size of maize but also lay a foundation for further fine mapping and even cloning of the promising loci.

## Introduction

Maize is an important agricultural crop. It can be served as food, animal feed, and industrial materials (Li et al., 2017) and plays a special role in food security (Liu et al., 2014). High grain yield has always been the most important goal of maize breeding. But most yield traits are quantitative traits controlled by multiple genes (Lynch and Walsh, 1998; Xu, 2010). KL and KW are both considered to be important yield traits (Doebley et al., 2006). Kernel size traits, especially KW, has been revealed to be significantly correlated with grain yield of maize (Li et al., 2013). The improvement of kernel size is therefore of great significance in maize breeding.

To date, numerous studies on maize grain yield traits have been reported at phenotypic levels (Rafiq et al., 2010; Nzuve et al., 2014). However, the genetic architecture and molecular mechanisms underlying natural quantitative variation in kernel yield have not been completely elucidated (Chen et al., 2016). Along with the first genetic linkage map of maize, published in 1986 (Helentjaris et al., 1986), molecular markers based on polymerase chain reaction (PCR) technology have greatly developed and applied for constructing genetic maps. Then, increasing QTLs controlling important agronomic traits in maize were detected by analyzing phenotypic value based on constructed genetic maps. These identified QTLs were distributed on all 10 maize chromosomes (Qiu et al., 2011). Many QTL mapping or fine mapping works for kernel size or weight have been carried out in recent years (Liu et al., 2014; Zhang et al., 2014; Chen et al., 2016). Till now, more than 150 QTLs for kernel size or weight have been identified by using different maize populations (Gramene QTL database). Liu et al. (2020) detected 50 QTLs for kernel size traits in the intermated B73 × Mo17 (IBM) Syn10 doubled haploid (DH) population, of which eight were repetitively identified in at least three environments. A total 55 and 28 QTL for kernel traits were identified by using composite interval mapping (CIM) for single-environment analysis and mixed linear model-based CIM for joint analysis, respectively, with 270 F_2__:__3_ families derived from the cross between V671 (large kernel) × Mc (small kernel)in five environments (Liu et al., 2014). It is critically important that QTLs should be validated and fine mapped for applying in further marker assisted breeding process. The near-isogenic line (NIL) is one of the most widely accepted populations commonly used for QTL fine mapping. NILs have been successfully used in confirming and fine-mapping QTLs in many species, such as rice (Lin et al., 2003; Li et al., 2004; Xie et al., 2006) and wheat (Xue et al., 2013; Zheng et al., 2015). In maize, a major QTL *qkrnw4* associated with kernel row number was mapped by using a NIL population (Nie et al., 2019). Gao et al. (2019) mapped *qLRI4*, which conferred leaf rolling index by using NIL populations. Yang et al. (2018) mapped a major QTL *qkc7.03* to a 416.27 kb physical interval for kernel cracking with NILs developed.

Great achievements in QTL mapping or isolating underlying genes for kernel size have been made in many species such as rice (Wan et al., 2006; Song et al., 2007; Li et al., 2011; Qiu et al., 2012; Kang et al., 2018), *Arabidopsis thaliana* (Xia et al., 2013; Du et al., 2014), soybean (Xu et al., 2011; Han et al., 2012), and wheat (Sun et al., 2009; Ramya et al., 2010). In particular, genes controlling rice kernel size or weight, such as *GS3* (Fan et al., 2006), *GS5* (Li et al., 2011), *qGL3* (Zhang et al., 2012), *GW2* (Song et al., 2007), *GW8* (Wang et al., 2012), *GS2* (Hu et al., 2015), *qGW7/GL7* (Wang et al., 2015), have been successfully cloned. The study of identifying and cloning kernel-size-related genes has lagged in maize. To a certain extent, the reason for this might be due to the genome of maize is large and complicated for many transposable elements and repetitive sequences exist (Gaut et al., 2000; Feuillet and Eversole, 2009). In addition, most complex traits, such as kernel yield and kernel size, are controlled by many genes with small effects (Edwards et al., 1987; Tian et al., 2011). QTLs identified in different genetic backgrounds across multiple environments have a higher chance of being positionally cloned (Chen et al., 2016).

Based on previous studies, the purposes of this study were as follows: (1) to map QTLs for kernel size in three environments by using F_2_ and F_2__:__3_ populations from the same cross SG5/SG7; (2) to validate and fine map the identified major QTL *qKL-2* by using BC_3_F_1_NILs; and (3) to reveal differently expressed genes (DEGs) between SG5 and SG7 by RNA-seq technology and predict candidate genes responsible for KL. In the study, we constructed an F_2_ and an F_2__:__3_ populations using two maize inbred lines SG5 and SG7 and evaluated them in three environments for mapping QTLs for kernel size. Furthermore, we finely mapped a major QTL by using the NILs from the cross of SG5 and SG7 and used RNA-seq technology to reveal the DEGs between parental lines SG5 and SG7. Finally, the candidate genes for *qKL-2* were predicted.

## Results

### Phenotype Evaluation for Segregation Populations

Two kernel size traits, i.e., KL and KW were estimated. The trait values of F_2_ population were investigated in 2016, while the phenotypic values of F_2__:__3_ populations were collected in 2018 and 2019, and these were recorded as F_2__:__3_-2018 and F_2__:__3_-2019, respectively. Table 1 presents the mean values of KL and KW investigated from F_2_ and F_2__:__3_ populations. The two inbred lines SG5 and SG7 were significantly different in both KL and KW traits. KL showed extremely significantly different between SG5 and SG7 (*P* < 0.01, Figures 3A,B). The data of two kernel size traits both emerged on normal distribution (Supplementary Figure 1). The two traits correlated positively with each other, with Pearson’s correlation coefficient being 0.20, 0.25, and 0.24 in F_2_-2016, F_2__:__3_-2018, and F_2__:__3_-2019, respectively.

**TABLE 1:** Descriptive statistics of KL and KW traits in the F_2_ and F_2__:__3_ mapping populations of maize derived from the cross between SG5 and SG7.

Generations	Trait	SG5 (mm)	SG7 (mm)	Min (mm)	Max (mm)	Mean (mm)	SD (mm)
F_2_	KL	9.93	8.99	8.07	12.87	10.42	0.85
	KW	8.07	11.17	8.03	11.80	9.98	0.78
F_2:__3_-2018	KL	9.93	8.99	8.49	13.21	10.34	0.74
	KW	8.07	11.17	8.24	12.12	10.07	0.74
F_2:__3_-2019	KL	9.93	8.99	8.40	13.35	10.34	0.75
	KW	8.07	11.17	8.28	12.22	10.08	0.73

### QTL Mapping

**FIGURE 1:**
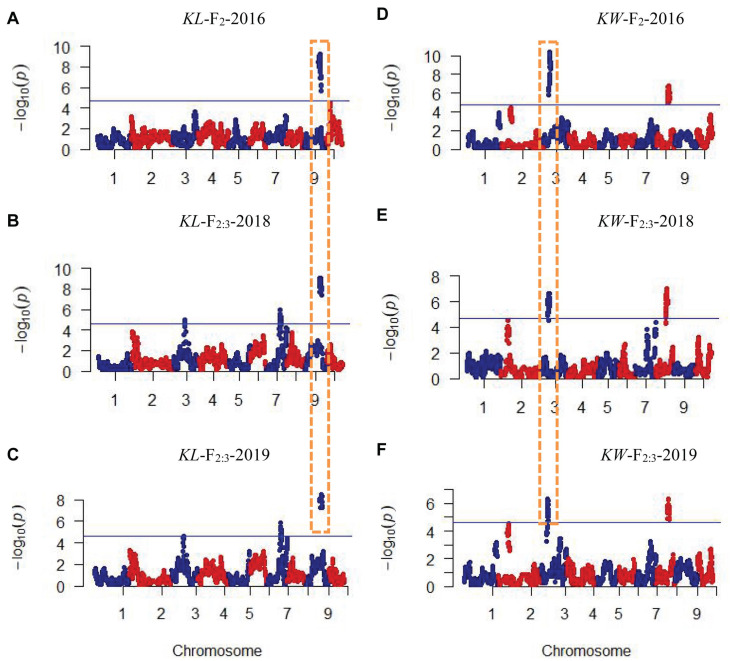
Plots of test statistic−Log10(p) against genome location for KL and KW traits in maize using the CIM method. The horizontal blue line of each panel is the critical value of the test statistic generated from 1,000 permuted samples. **(A–C)** Indicate KL mapping results in three environments while **(D–F)** indicate KW mapping results in three environments. Dotted rectangles with orange color indicate these QTLs were mapped repeatedly in all three environments.

**FIGURE 2:**
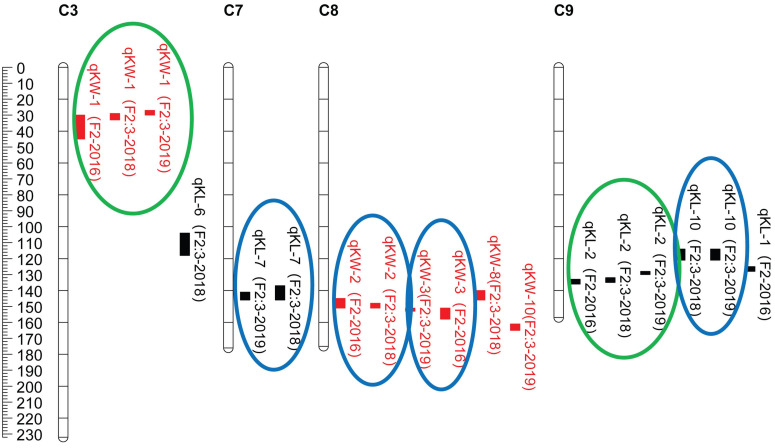
QTL locations for KL and KW traits studied in the F_2_ and F_2:3_ populations from cross SG5/SG7. QTLs were represented in different colors for kernel size traits including red for KW (kernel width, mm), black for KL (kernel length, mm) on maize chromosomes 3, 7, 8, and 9. QTLs represented by bars are shown on the right of the linkage groups close to their corresponding markers. Supported intervals for each QTL are indicated by the length of vertical bars. The QTLs circled in green were stably detected in three environments while QTLs circled in blue were repeatedly detected in two environments.

CIM procedure was applied to map QTLs conferring KL and KW. Manhattan plots were shown in Figure 1. A total of 10 QTLs were mapped in total for KL and KW from F_2_ and F_2__:__3_ populations. The information is summarized in Table 2. For KL, two major QTLs were mapped on maize chromosome 9 in F_2_ population. A total four QTLs were mapped on chromosomes 3, 7, and 9 in F_2__:__3_-2018 population while three QTLs were mapped on chromosomes 7 and 9 in F_2__:__3_-2019 population. For KW, three QTLs were mapped on maize chromosomes 3 and 8. A total three QTLs were mapped on maize chromosomes 3 and 8 in both F_2__:__3_-2018 and F_2__:__3_-2019 populations, respectively. The phenotypic variation explained by these QTLs ranged from 8.4 to 23.0%, with a mean value of 14.25 and 14.46%, 14.03 and 12.97%, and 10.83 and 13.67% for KL and KW in F_2_-2016, F_2__:__3_-2018, and F_2__:__3_-2019, respectively. The LOD score ranges from 4.0 for *qKL-7* to 9.5 for *qKW-1*. Among the 10 QTLs, *qKL-2* for KL, and *qKW-1* for KW were detected in all the three environments (Figure 2, highlighted in green color circle). That is, they were stable QTLs in the study. Four QTLs (*qKW-2*, *qKL-7*, *qKW-3*, and *qKL-10*) were detected in two environments, highlighted in blue color circle in Figure 2. In addition, three QTLs, *qKL-1*, *qKW-1*, and *qKW-2*, overlapped with the QTLs identified from the metaQTL analysis (Chen et al., 2017).

**TABLE 2:** QTL identified for KL and KW traits of maize using high-density SNP bin-map from composite interval mapping (CIM).

Environments	QTL	Chr	Flanking markers	Positions (Mb)	Interval (Mb)	Physical length (Mb)	LOD^*c*^	ADD^*a*^	DOM^*b*^	*R*^2^ (%)
**Kernel length (KL) trait**									

F_2_-2016	***qKL-1***	**9**	**mk3093–mk3100**	**126.05**	**124.90–127.55**	**2.65**	**8.4**	**0.34**	**0.18**	**14.8**
	***qKL-2***	**9**	**mk3106–mk3114**	**134.65**	**133.20–135.75**	**2.55**	**7.7**	**0.26**	**0.24**	**13.7**
	*qKL-3*	3	mk1343–mk1350	217.85	215.90–218.1	2.20	2.9	−0.13	−0.17	4.7
	*qKL-4*	2	mk624–mk638	2.75	0.10–3.55	3.45	2.5	0.06	0.254	4.2
	*qKL-5*	10	mk3228–mk3236	105.05	96.45–109.2	12.75	3.8	−0.19	−0.14	6.4
F_2__:__3_-2018	***qKL-2***	**9**	**mk3105–mk3110**	**134.65**	**131.55–134.75**	**3.20**	**8.1**	**0.23**	**0.25**	**15.3**
	*qKL-4*	2	Mk624–mk634	2.85	0.10–3.00	2.90	3.1	−0.02	0.31	5.4
	***qKL-6***	**3**	**mk1145–mk1167**	**115.20**	**103.65–117.85**	**14.2**	**4.2**	−**0.38**	**0.17**	**12.1**
	***qKL-7***	**7**	**mk2510–mk2525**	**143.00**	**137.30–145.70**	**8.40**	**5.1**	−**0.40**	**0.32**	**13.4**
	*qKL-8*	7	mk2618–mk2622	174.6.	174.35–174.90	0.55	3.5	−0.28	0.04	6.5
	*qKL-9*	8	mk2689–mk2701	21.85	21.60–33.80	12.20	3.1	−0.30	0.20	8.0
	***qKL-10***	**9**	**mk3077–mk3084**	**115.15**	**113.65–120.70**	**7.05**	**8.1**	**0.30**	**0.19**	**15.3**
F_2__:__3_-2019	***qKL-2***	**9**	**mk3100–mk3104**	**128.0.**	**127.55–130.05**	**2.50**	**6.9**	**0.27**	**0.17**	**13.4**
	*qKL-4*	2	mk624–mk637	2.85	0.10–3.45	3.35	2.6	0.00	0.27	4.5
	*qKL-6*	3	mk1158–mk1167	115.2.	114.80–117.85	3.05	3.9	−0.37	0.19	11.3
	***qKL-7***	**7**	**mk2517–mk2525**	**143.00**	**141.25–145.70**	**4.45**	**4.0**	**0.40**	**0.34**	**13.0**
	*qKL-8*	7	mk2617–mk2622	174.60	174.05–174.90	0.85	3.7	−0.26	-0.01	5.9
	***qKL-10***	**9**	**mk3077–mk3084**	**115.15**	**113.65–120.70**	**7.05**	**7.6**	**0.28**	**0.20**	**14.6**
	*qKL-11*	5	mk2183–mk2188	215.30	214.55–215.55	1.00	2.6	0.25	−0.26	5.9
	*qKL-12*	6	mk2298–mk2303	149.80	143.05–157.50	14.45	2.5	0.09	0.24	4.4

**Kernel width (KW) trait**										

F_2_-2016	***qKW-1***	**3**	**mk1042–mk1060**	**30.85**	**30.20–44.55**	**14.35**	**9.5**	−**0.54**	**0.17**	**23.0**
	***qKW-2***	**8**	**mk2806–mk2814**	**148.95**	**144.55–151.00**	**6.45**	**5.8**	−**0.34**	−**0.02**	**10.0**
	***qKW-3***	**8**	**mk2814–mk2820**	**152.25**	**151.00–157.95**	**6.95**	**5.9**	−**0.35**	**0.00**	**10.9**
	*qKW-4*	1	mk577–mk603	292.20	288.45–295.55	7.10	3.1	−0.29	0.09	7.2
	*qKW-5*	2	mk667–mk673	17.05	13.60–17.55	3.95	3.4	−0.23	−0.07	4.5
	*qKW-6*	2	mk676–mk686	24.05	19.55–24.35	4.80	3.7	−0.19	−0.13	6.1
	*qKW-7*	10	mk3284–mk3294	145.50	144.70–146.95	2.25	3.0	−0.15	0.33	4.9
F_2__:__3_*-*2018	***qKW-1***	**3**	**mk1040–mk1047**	**30.85**	**29.30–32.75**	**3.45**	**5.8**	−**0.37**	−**0.04**	**9.9**
	***qKW-2***	**8**	**mk2811–mk2814**	**148.95**	**147.80–151.00**	**3.20**	**5.8**	−**0.43**	**0.13**	**14.5**
	*qKW-5*	2	mk667–mk676	16.70	13.60–19.55	5.95	3.2	−0.12	−0.20	5.3
	***qKW-8***	**8**	**mk2799–2808**	**141.80**	**140.30–145.60**	**5.30**	**6.2**	−**0.43**	**0.11**	**14.5**
	*qKW-9*	2	mk657–mk662	9.70	9.30–11.30	2.00	3.8	−0.06	−0.29	6.2
F_2__:__3_*-*2019	***qKW-1***	**3**	**mk1035–mk1042**	**28.95**	**27.10–30.20**	**3.10**	**5.5**	−**0.47**	**0.19**	**14.5**
	***qKW-3***	**8**	**mk2814–mk2817**	**152.25**	**151.00–153.30**	**2.30**	**5.5**	−**0.33**	−**0.01**	**9.6**
	*qKW-4*	1	mk579–mk591	292.55	289.45–293.25	3.80	2.5	−0.23	0.05	5.0
	*qKW-6*	2	mk676–mk686	23.75	19.55–24.35	4.80	3.8	−0.17	−0.14	6.2
	***qKW-10***	**8**	**mk2826–mk2835**	**163.25**	**160.70–164.95**	**4.25**	**5.0**	−**0.24**	−**0.14**	**8.4**

*^a^Estimated additive effect. ^b^Estimated dominance effect. ^c^The logarithm of odds (LOD) value 3.86 for detected traits was set up by 1,000 times permutations results (in bold). QTL statistics were also reported for those in which the LOD score exceeded 2.5 but was less than 3.86 (in no bold).*

### Fine Mapping qKL-2 With NILs

**FIGURE 3:**
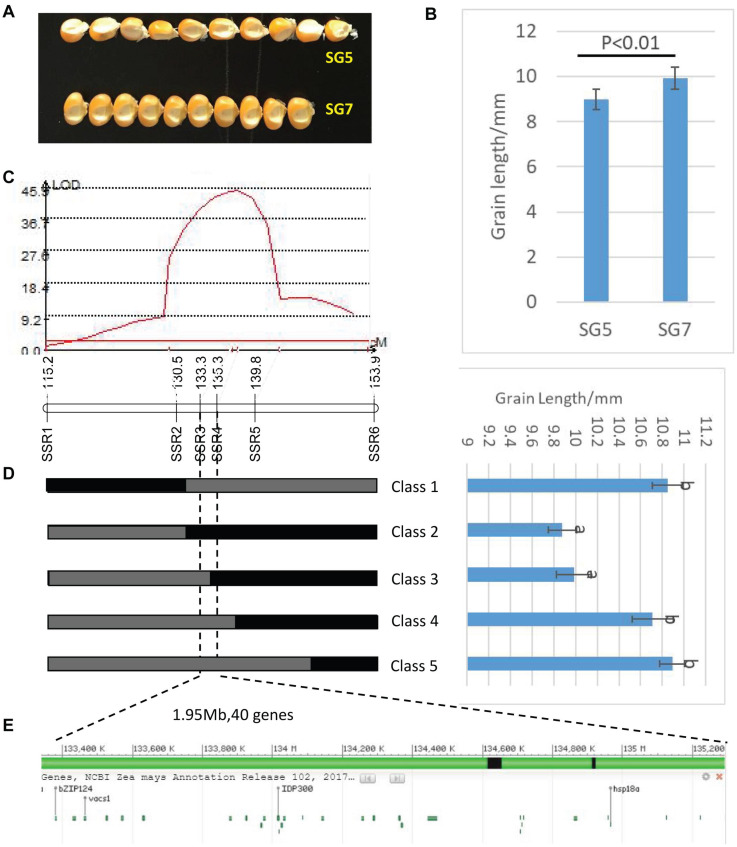
**(A)** Grain length difference between SG5 and SG7. **(B)** Significance test of difference between SG5 and SG7, *P* < 0.01 means that difference was extremely significant between SG5 and SG7. **(C)** LOD profile of *qKL-2*, which was identified in the BC_3_F_1_ populations. **(D)** Major QTL*qKL-2* was mapped in SSR3 and SSR5interval by using 998 BC_3_F_1_ plants. Class 1–Class 5 indicates the recombinants with different recombination types. Graphical genotypes and their phenotypic values of recombinant lines separated in BC_3_F_1_. Black bars and gray bars represent the chromosomal segments for the homozygous SG7 and heterozygous alleles, respectively. The progeny test of homozygous segregants indicated that *qKL-2* was located at an interval of 1.95 Mb and flanked by SSR3 and SSR4. Different letters indicate significant difference at the 0.05 level. **(E)** Physical positions of the 40 protein coding genes in the mapped 1.95 Mb region (B73 RefGen_v4).

From 2017 to 2019, a NIL population, consisting of 998 BC_3_F_1_ lines, was developed by introgressing the *qKL-2* genomic region of SG5 into the SG7 genetic background. A secondary linkage map with six markers (Supplementary Table 1) around *qKL-2* was generated. The six markers were located at 115.23, 130.51, 133.34, 135.29, 139.75, and 153.88 Mb on chromosome 9 by blasting maize B73 RefGen_v4 (Figure 3C). The secondary linkage map was 43.35 cM in length, and the genetic distances between every two adjacent markers were 16.75, 8.39, 0.80, 5.67, and 11.74 cM.

Then the major QTL *qKL-2* was detected with the secondary linkage map of NILs by CIM method in QTL Cartographer v2.5. The *qKL-2* had an additive effect of 0.97 mm and explained 16% of phenotypic variation. The LOD peak indicated that *qKL-2* was most likely located between SSR3 and SSR5, the LOD peak position was located between SSR3 and SSR4 (Figure 3C). To confirm the narrowed *qKL-2* interval, five recombinant types, namely, Class 1–Class 5, were selected from 998 NILs. Class 1 indicates that 28 recombinants with SSR1 and SSR2 homozygous and SSR3–SSR6 heterozygous. Class 2 indicates 33 recombinants with SSR1 and SSR2 heterozygous and SSR3–SSR6 homozygous. Class 3 indicates three recombinants with SSR1–SSR3 heterozygous and SSR4–SSR6 homozygous. Class 4 indicates 20 recombinants with SSR1–SSR4 heterozygous and SSR5–SSR6 homozygous. Class 5 indicates 47 recombinants with SSR1–SSR5 heterozygous and SSR6 homozygous. At SSR3 and SSR4 loci, Classes 2 and 3 were homozygous while Classes 1, 4, and 5 were heterozygous. There was significantly difference in phenotypic values between the two set of recombinants Classes 2 and 3 and Classes 1, 4, and 5 (Figure 3D). The progeny test of homozygous segregants indicated that *qKL-2* was located in an interval of 1.95 Mb (133.34–135.29) and flanked by SSR3 and SSR4 physical interval (Table 3). The selected overlapping recombinant chromosomes also supported the location of *qKL-2*.

**TABLE 3:** Statistical analysis of phenotypic values from different types of recombinants in NILs around the *qKL-2* region.

	No. of recombinants	Grain length/mm
Class^*a*^ 1	28	10.85 ± 0.14
Class 2	33	9.88 ± 0.13
Class 3	3	9.99 ± 0.16
Class 4	20	10.71 ± 0.19
Class 5	47	10.89 ± 0.11

*^a^Class 1–Class 5 indicates different types of recombinants with six SSR markers. Class 1 indicates that 28 recombinants with SSR1 and SSR2 homozygous and SSR3–SSR6 heterozygous. Class 2 indicates 33 recombinants with SSR1 and SSR2 heterozygous and SSR3–SSR6 homozygous. Class 3 indicates three recombinants with SSR1–SSR3 heterozygous and SSR4–SSR6 homozygous. Class 4 indicates 20 recombinants with SSR1–SSR4 heterozygous and SSR5–SSR6 homozygous. Class 5 indicates 47 recombinants with SSR1–SSR5 heterozygous and SSR6 homozygous.*

### Candidate Genes for qKL-2 Prediction

RNA-seq procedure was conducted for 18 RNA grain samples at different developmental stages. Results showed that the 1.95 Mb physical intervals of *qKL-2* encompassed 40 protein coding genes (Figure 3E). After DEGs analysis, a total of 11 protein coding genes differently expressed and left in the *qKL-2* physical intervals (Table 4). Previous studies indicated that FERONIA receptor kinase controls seed size in *Arabidopsis thaliana* (Yu et al., 2014). GRMZM2G006080 encodes receptor-like protein kinase FERONIA and was predicted as a candidate gene of *qKL-2*, which is most likely responsible for KL.

**TABLE 4:** Differentially expressed genes out of 40 protein coding genes in 1.95 Mb physical interval on chromosome 9 and candidate gene predicted for *qKL-2*.

GeneID (B73 RefGen_v3)	Start (bp)	End (bp)	Length	Annotation from blast swiss prot	LogFC^*a*^ or RCP1/RCP2^*b*^		
					Day 5^*c*^	Day 10	Day 15
GRMZM2G099101	132,128,943	132,133,712	2,349	Endoglucanase 9	0.36	0.42	1.66
GRMZM5G899188	132,973,437	132,974,250	814	17.0 kDa class II heat shock protein	2.03	0.98	3.23
GRMZM2G398288	131,627,588	131,635,485	4,521	Phospholipid-transporting ATPase 1	−0.40^*d*^	0.36	−0.22
GRMZM2G027437	131,778,230	131,786,437	1,277		−0.55	−0.45	−0.79
GRMZM2G006080	131,829,772	131,832,943	3,172	Receptor-like protein kinase FERONIA	0.41	−0.09	−0.04
GRMZM2G309327	131,924,112	131,925,141	1,030	Probable calcium-binding protein	−1.87	−2.20	−1.41
GRMZM2G159500	133,066,105	133,068,297	2,048	NAC domain-containing protein	−0.76	-0.22	−0.21
GRMZM2G102382	133,137,426	133,140,041	1,413	Thioredoxin-like 1–2, chloroplastic	0.85	0.95	1.01
GRMZM2G102657	13,279,7892	132,800,350	2,053		0/9.54	0/18.55	0/7.55
GRMZM2G404249	132,803,660	132,804,501	842	17.0 kDa class II heat shock protein	1.70	2.29	1.27
GRMZM5G875954	132,964,847	132,972,815	1,206	Selenium-binding protein 2	−5.07	−3.20	−1.20

*^a^Log 2 ratio, number of folds the gene is differentially expressed in RNA-seq. ^b^Different of read counts between P_1_ and P_2_. ^c^Day 5, day 10, day 15 indicate grain samples collected after selfing 5, 10, and 15 days between the two parents SG5 and SG7. ^d^Positive sign indicates gene transcript expressed high in SG5 while negative sign indicates gene transcript expressed high in SG7.*

## Discussion

Kernel size controlled by multiple genes is an important component of grain yield in maize. Grain yield was influenced significantly by kernel size, especially KL (Li et al., 2009, 2013). Stable QTLs are of great significance for marker-assisted breeding, while false positive QTLs are of no use. Normally, two steps, i.e., primary mapping and fine mapping, are needed for QTL analysis unless experiments were conducted in multiple environments with as many as sample size and marker numbers. In this study, primary mapping was carried out in three environments, and two kernel-size QTLs, *qKL-2*, and *qKW-1*, detected in all three environments were stable. The two QTLs could be benefit for further marker assisted breeding. Chen et al. (2017) conducted metaQTL analysis based on collecting information on QTLs conferring maize yield-related traits from 33 published studies. A total of 76 MQTLs for maize yield and its related traits were identified across the whole genome, with the number per chromosome ranging from four on chromosome 4–10 on chromosome 5 (Chen et al., 2017). After comparing with the metaQTL analysis results, *qKL-1, qKW-1*, and *qKW-2* detected in this study all overlapped with those MQTLs for kernel-related traits but with more decreased physical intervals (Table 2).

For *qKL-2* locus, primary mapping results showed that the physical intervals were 133.20–135.75, 131.55–134.75, and 127.55–130.05 Mb on chromosome 9, respectively, in three environments. In order to confirm and fine map *qKL-2*, a NIL population was developed by continuous backcross with markers assisted selection for confirming and fine mapping *qKL-2*. Finally, *qKL-2* was mapped in a 1.95 Mb (133.34–135.29 Mb) interval on maize chromosome 9. Compared with metaQTL analysis results from Chen et al. (2017), MQTL-66, which includes 16 QTLs related to grain yield, ear-related traits, and kernel-related traits located in 120.2–133.6 physical interval on chromosome 9. There was only 0.26 Mb physical distance overlap for *qKL-2* (133.34–135.29 Mb) and MQTL-66 (120.2–133.6 Mb). It is very likely that *qKL-2* was a new locus to control KL.

It is of critical importance that the less genes the better in target QTL interval for map-based cloning. In this study, RNA-seq technology was applied for transcriptomic analyzing DEGs between SG5 and SG7 grains in different developmental stages. DEGs identified were located in the *qKL-2* interval. After DEGs analysis, only 11 protein coding genes were left in the QTL *qKL-2* intervals (Table 4). The potential functional genes in QTLs physical intervals decreased significantly after DEGs analysis. According to gene annotation from Blast swiss prot, the function of 11 genes include endoglucanase, 17.0 kDa class II heat shock protein, phospholipid-transporting ATPase 1, receptor-like protein kinase FERONIA, calcium-binding protein, selenium-binding protein 2, NAC domain-containing protein, and thioredoxin-like 1–2, chloroplastic. Further comparative genomics analysis was applied for predicting candidate genes. The evidence on studies of rice or *Arabidopsis thaliana* showed that kernel size was regulated by multiple signaling pathways, including ubiquitin-proteasome degradation (Verma et al., 2004), the transcription factor pathway, the phytohormone signaling pathway, and the G protein independent pathway. Yu et al. (2014) concluded that receptor kinase FERONIA involved in a signaling pathway negatively regulated the elongation of integument cells and then controlled the seed size in *A. thaliana*. Based on the above function analysis of 11 protein coding genes, GRMZM2G006080, which encodes receptor-like protein kinase FERONIA, was predicted as a candidate gene of kernel size. The predicted candidate gene will not only be helpful for underlying genetic mechanism for kernel size but also provides a basis for improving kernel size traits in maize.

## Materials and Methods

### Segregation Population Development and Phenotypic Evaluation

Two maize inbred lines, SG5 and SG7, were used in the study. The seeds were provided by the Institute of Grain and Oil, Liupanshui Academy of Agricultural Sciences, Liupanshui, China. We developed an F_2_ population by crossing SG5 and SG7 in Liupanshui, Guizhou province of China in the summer of 2013 and 2014. A total of 199 F_2_ individuals were planted at the Panxian Maize Breeding Station in Sanya, China, in the winter of 2014. Then, an F_2__:__3_ segregation population containing 199 lines was developed by selfing each F_2_ individuals. The F_2__:__3_ population was planted at the Panxian Maize Breeding Station in Sanya for kernel size evaluation in the summer of 2018 and 2019. Field experiment was performed in a randomized block design with three replications. Single-row plots with row spacing of 50 cm were adopted, and each plot grew 15 plants with plant spacing of 35 cm. Kernel size traits, including KL and KW, were investigated in both F_2_ and F_2__:__3_ populations after corns were harvested and dried naturally. For F_2_ generation, the traits were estimated by the mean value of three repeats including 10 kernels of an ear. For F_2__:__3_, kernel size evaluation was based on eight ears from the middle part of each plot. KL and KW were estimated by mean value of three repeats including 10 kernels randomly selected from bulked kernels of eight ears. The measured kernels were all sampled from the middle part of an ear.

Young leaves were collected from each F_2_ individual for DNA extraction. The methods of genomic DNA extraction, genotype sequencing, and grouping, single nucleotide polymorphisms (SNPs) identification, and high-density linkage map construction were presented in our previous study (Su et al., 2017). The forward regression model of CIM method in QTL Cartographer v2.5 was applied for QTL mapping with walking speed of 1 cM. The likelihood of odds (LOD) value 3.86 was used to declare a QTL, which was based upon 1,000 times permutations analysis. QTL statistics were also reported for those in which the LOD score exceeded 2.5. LOD peaks were used for determining the position of a significant QTL on chromosomes. The positive additive effect value of a QTL indicates that the increase in phenotypic value is provided by SG5 alleles while negative value indicates the decrease in phenotypic value is provided by SG7 alleles. MapChart 2.32 software (Voorrips, 2002) was used for the graphical presentation of QTLs. The QTLs that are mapped in F_2_ and F_2__:__3_ populations were compared, and the consistent one will be regarded as stable QTL.

### NILs Development and qKL-2 Fine Mapping

NILs for the *qKL-2* locus were developed by using continuous backcrossing combined with marker-assisted selection methods. The SSR molecular markers that are near *qKL-2* and are polymorphic between donor parent SG5 and recurrent parent SG7 were used for marker-assisted selection of the BC_2_F_1_ generation. These SSR markers, based on resequencing maize genome results, were all developed by Xu et al. (2013). We choose SSRs that are near *qKL-2* position with high polymorphism information content (PIC) values. These SSRs were used for screening polymorphism between our parental lines SG5 and SG7. SSRs with clearly bands of polyacrylamide gel electrophoresis (PAGE) and polymorphism between SG5 and SG7 were selected for developing secondary linkage map and further fine mapping works. Phenotypic value for BC_3_F_1_ lines was investigated in the same way as for the F_2_ population. Young healthy leaves were collected from each of the 998 BC_3_F_1_ line for genomic DNA extraction. Plant Genomic DNA Kit (TIANGEN, Beijing, China) were used and the manufacturer’s protocols were followed. DNA purity was checked by 1% agarose gel and NanoPhotometer R spectrophotometer (IMPLEN, CA, United States). DNA concentration was then measured using an Qubit R DNA Assay Kit in Qubit R 2.0 Flurometer (Life Technologies, CA, United States).

The secondary linkage map around *qKL-2* was generated by JoinMap 3.0 software (Van Ooijen and Voorrips, 2001). QTL Cartographer v2.5 was applied for QTL mapping with the CIM method, walking speed 1 cM, and a LOD threshold of 10.0.

### Candidate Gene for qKL-2 Prediction

Grains of SG5 and SG7 were sampled on the 5th, 10th, and 15th days after selfing three biological replicates. All collected samples were immediately frozen in liquid nitrogen and then transferred to a −80°C environment before RNA extraction. We finally got 18 grain samples in total. All the samples were sequenced at the Illumina NovaSeq platform. Raw reads with fastq format were firstly handled by in-house perl scripts. Clean reads were then obtained after deleting reads containing adapters and ploy-N and removing reads of a low quality in raw data. In the meantime, the GC content and Q20 and Q30 of the clean reads were calculated. High-quality clean data were then carried out for further downstream analyzing. Reference genome was downloaded directly from genome website^1^, and correlated files of gene annotation were also downloaded from the same website. Bowtie v2.2.3 was used for building reference genome index and TopHat v2.0.12 (Trapnell et al., 2013) was used for aligning paired-end clean reads to the reference genome. The number of reads mapped to each gene was counted by HTSeq v0.6.1. For each gene, the expected number of fragments per kilobase of transcript sequence per millions base pairs (FPKM) was calculated by analyzing the gene length and reads mapped to the gene. FPKM is a widely accepted method currently to evaluate levels of gene expression based on considering sequencing depth effect and gene length of the read count simultaneously (Trapnell et al., 2010). The DEGSeq R package (1.20.0) was applied for analyzing differential expression between two conditions. The *P*-values adjusted by using the Benjamini and Hochberg method were used. The threshold of corrected *P*-value 0.005 and log 2 (Fold change) of 1 (absolute value) were considered as significantly differential expression. More information about the methods for reference genome index construction, paired-end clean reads alignment and count, FPKM calculation and DEGs analysis referred to our previous study (Zhao and Su, 2019).

Through analyzing DEGs between SG5 and SG7, the DEGs that were overlaid on to a physical interval of *qKL-2* were considered as candidate genes for kernel size in maize. The detected DEGs were further annotated from Blast Swiss Prot database.

## Data Availability Statement

The datasets generated for this study can be found in NCBI BioProject PRJNA673992, https://www.ncbi.nlm.nih.gov/bioproject/PRJNA673992/. We have uploaded our SNP sequencing data to FigShare repository https://doi.org/10.6084/m9.figshare.13120322.v1.

## Author Contributions

GW and YZ developed the F_2_, F_2__:__3_, and BC_3_F_1_ population. WM and XM performed the phenotype investigation. CS developed the genotyping of F_2__:__3_ progeny, analyzed the data, and drafted the manuscript. All authors read and approved the final manuscript.

## Conflict of Interest

The authors declare that the research was conducted in the absence of any commercial or financial relationships that could be construed as a potential conflict of interest.

## Footnotes

1 ftp://ftp.ensemblgenomes.org/pub/plants/release-29/fasta/zea_mays/dna/Zea_mays.AGPv3.29.dna.toplevel.fa.gz
