# Supplementary material for: QTL Analysis and Fine Mapping of a Major QTL Conferring Kernel Size in Maize (Zea mays)
Source: Front Genet. 2020 Nov 27;11:603920. doi: 10.3389/fgene.2020.603920 (PMC7728991; doi:10.3389/fgene.2020.603920)
Supplement: Supplementary file 1 [file Table_1.DOCX]

**Supplemental Table 1.** List of primers used in this study

| Name | Forward | Reverse |
| --- | --- | --- |
| SSR1 | 5’-GCGATGCTGGTAGTTGGACT | 5’-GCATGCACGCCTAATCTTCG-3’ |
| SSR2 | 5’-CGGGCATCCATATCCCAACA | 5’-GGAGCCTACTACGCCATGAC-3’ |
| SSR3 | 5’-ATTAAGAGTGACTGCCCCGC | 5’-TCCAGAGACGTCGAGGAAGA-3’ |
| SSR4 | 5’-TTCAAGTCCGATAGCGAGGC | 5’-GAGGCAAAGCAACGCTGAAT-3’ |
| SSR5 | 5’-CCTACCGCTACTGCTGCTAC | 5’-GATGTCTGTGGCTTGGGTCA-3’ |
| SSR6 | 5’-GTATGGGAGCAAGCTCGACA | 5’-CTCCTGCTCGAGTCCTCTCT-3’ |
