# Supplementary material for: QTL Analysis and Fine Mapping of a Major QTL Conferring Kernel Size in Maize (Zea mays)
Source: Front Genet. 2020 Nov 27;11:603920. doi: 10.3389/fgene.2020.603920 (PMC7728991; doi:10.3389/fgene.2020.603920)
Supplement: Supplementary file 2 [file Data_Sheet_1.docx]

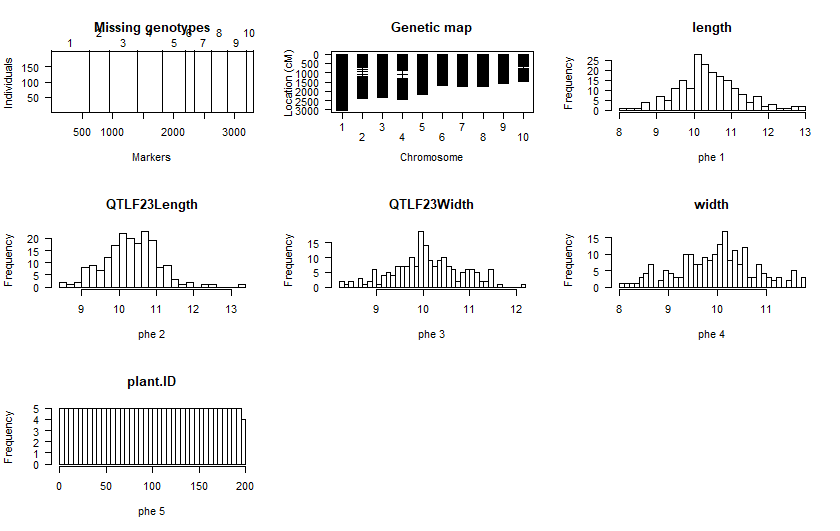

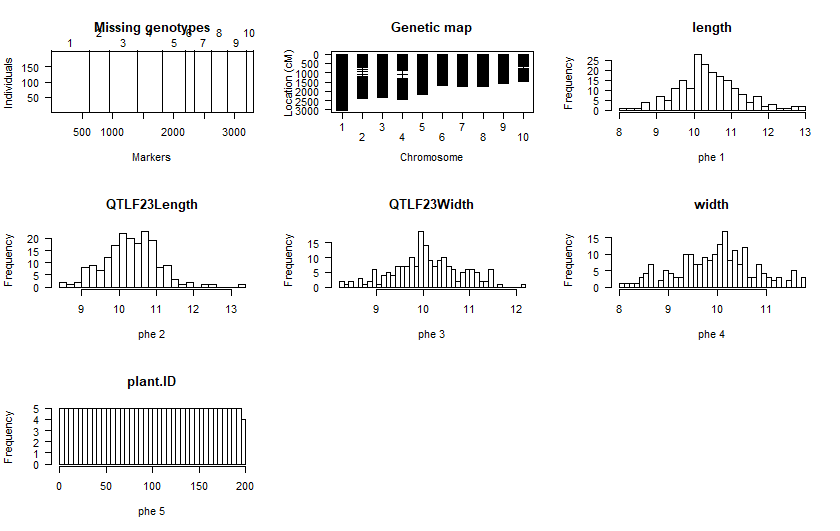

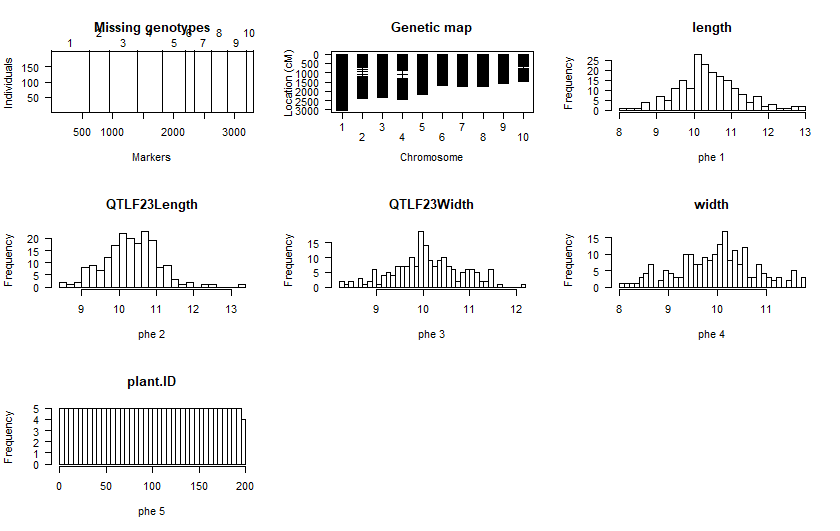


a

b

c

d

**Supplemental Fig.1.** Distribution of kernel length (*KL*), kernel width(*KW*) traits in F_2_ and F_2:3_ population derived from the cross of SG5 and SG7. Figure a, c and e indicate *KL* distribution while Figure b, d and f indicate *KW* distribution in three environments i.e., F_2_-2016, F_2:3_-2018 and F_2:3_-2019.


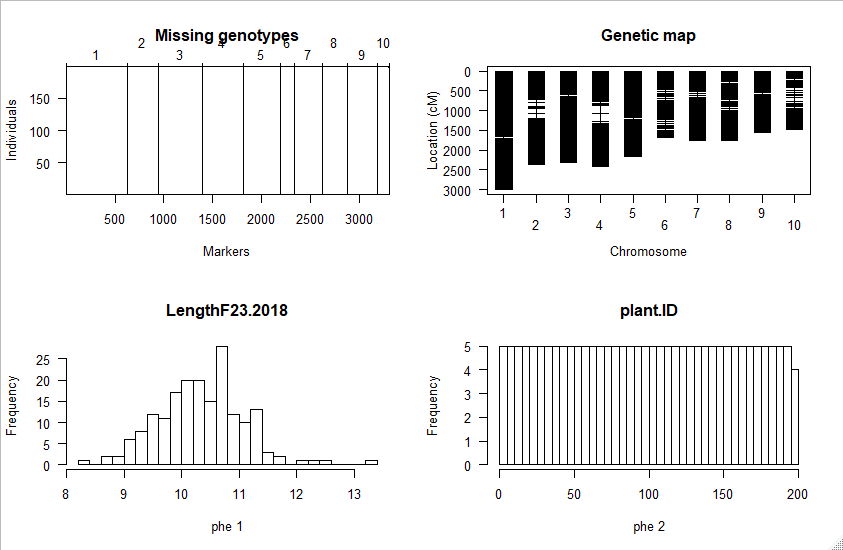

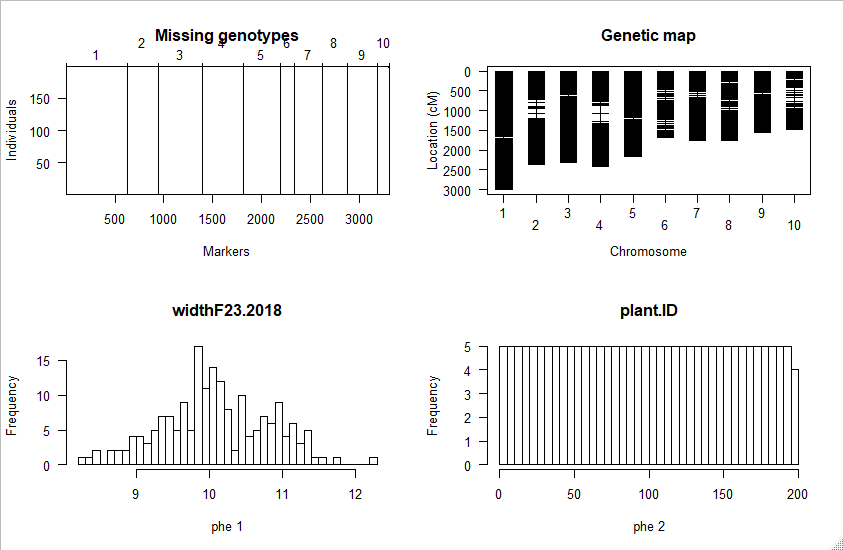


e

f
